# Cortical Circuitry and Synaptic Dysfunctions in Alzheimer's Disease and Other Dementias

**DOI:** 10.1155/2021/9796576

**Published:** 2021-07-29

**Authors:** Federico Ranieri, Alberto Benussi, Mariagiovanna Cantone, Florinda Ferreri, Javier Márquez-Ruiz

**Affiliations:** ^1^Unit of Neurology, Department of Neuroscience, Biomedicine and Movement Sciences, University of Verona, Verona, Italy; ^2^Unit of Neurology, Department of Clinical and Experimental Sciences, University of Brescia, Italy; ^3^Department of Neurology, Sant'Elia Hospital, ASP Caltanissetta, Caltanissetta, Italy; ^4^Unit of Neurology and Neurophysiology, Department of Neuroscience, University of Padova, Padova, Italy; ^5^Department of Physiology, Anatomy and Cell Biology, Pablo de Olavide University, Seville, Spain

A main challenge of current research on primary degenerative dementia is establishing a causal and temporal link between structural alterations, synaptic and circuit dysfunction, and cognitive decline. Experimental data support the view that the observable symptoms of dementia are only the tip of an iceberg, made up of neuropathological alterations which accumulate over time and lead to loss of synaptic connections and dysfunction of cortical circuitry [1].

In the clinical setting, current biomarkers are mainly based on the detection of amyloid deposition and neuronal injury. Increasing evidence also suggests a role for functional neuroimaging and electrophysiological parameters, derived from functional MRI (fMRI), electroencephalography (EEG), and transcranial magnetic stimulation, as biomarkers of dysfunction of neurotransmission and synaptic plasticity in specific types of primary dementia [2–6].

By considering the two major proteinopathies underlying Alzheimer's disease (AD), amyloid beta (A*β*) dysregulation, and Tau phosphorylation, the initial view of A*β* peptide accumulation acting as a key upstream event leading to neurodegeneration was put under revision since it has been acknowledged that these two neuropathological processes might be initiated independently [7, 8]. Indeed, while amyloid accumulation can progress over more than two decades [9], A*β* markers are not strongly related to cognitive decline as markers of neurodegeneration [1]. Significantly, in sporadic AD, impairment of long-term potentiation- (LTP-) like cortical plasticity was found to be associated with a higher cerebrospinal fluid total-Tau level and with a faster progression of cognitive decline [6].

In this effort to establish a link with functional impairment, in AD, attention has long been focused on the central cholinergic deficit and impairment of synaptic plasticity, but recent findings also point towards different neurotransmitter systems, prominently towards selective dopaminergic neuron degeneration leading to hippocampal LTP dysfunction as a possible upstream phenomenon [10]. A primary dysfunction of synaptic plasticity, possibly favored by genetic factors, has also been hypothesized as the leading cause of molecular and histopathological modifications [11].

A better understanding of such alterations is required for the development of drugs that target specific pathological mechanisms. Moreover, the early detection of pathological changes is crucial for the use of novel disease-modifying treatments that need to be administered early in the disease course [12].

The present Special Issue collects nine articles that contribute to the investigation of cortical circuit and synaptic dysfunctions related to molecular alterations.

Within the debate on the relationship between A*β* and Tau pathology, S. Mondragón-Rodríguez et al. review evidence supporting an alternative hypothesis on the role of Tau phosphorylation as a possible neuroprotective rather than neurodegenerative mechanism, acting by preventing synaptic overexcitation during the early stages of AD development. In this article, the authors propose that phosphorylation of Tau's microtubule domain is a fundamental part of the regulatory mechanisms controlling the activity of NMDA glutamate receptors and synaptic coupling. The presented hypothesis has important implications for current therapeutic approaches aimed at reducing Tau and phosphorylated Tau (pTau) levels. Further knowledge on the link between A*β* and Tau/pTau at the synapse is needed to support the hypothetic non-pathological role of pTau.

M. A. Aguiar-Furucho and F. J. R. Peláez simulated the effects of brain aging, so as to reproduce early AD stages, on a computational model of cortical circuits (of somatosensory, visual, and auditory cortices) involved in the first level of processing of sensory afferent inputs. The model, which has learning capabilities, includes cholinergic excitatory projections to thalamo-cortical neurons and inhibitory GABAergic interneurons. Within this model, the authors show that, in the presence of impaired GABA-A signaling, which has been described in AD pathophysiology, the reduction of afferent inputs determines a dysregulation of neuronal excitability that may facilitate a state of hypermetabolism. While the above model is focused on function rather than on a specific biological substrate, it provides a hypothesis that sensory/cognitive stimulus reduction might not only facilitate dementia by weakening cognitive reserve but also represent a direct pathophysiological mechanism of neurodegeneration.

J. Li et al. provide an overview of the GABAergic circuitry, and they highlight the mechanisms through which hippocampal GABAergic impairment may cause cognitive deficits under an ischemic environment, particularly at chronic ischemic stages. They review evidence of ischemia-induced changes of the GABAergic system, which has a significant role in promoting neural development and formation of local neural circuits, including GABA interneurons, extracellular GABA neurotransmitters, and GABA receptors. Moreover, to bring new insights and strategies to solving this kind of emerging cognitive deficits, they also describe exogenous cell transplantation technology, which is effectively supposed to improve cognition by modulating the GABAergic system.

As far as the A*β* is concerned, C. Gauthier-Umaña et al. investigated alterations of synaptic activity and plasticity in an animal model of early A*β* pathology. Results show that soluble A*β* microinjection in the commissural CA3-to-CA1 circuit increased synaptic variability, impaired long-term plasticity, increased gamma and high-frequency oscillations (121-250 Hz), and induced a significant *γ* amplitude-modulating *θ* phase shift in anaesthetized Wistar rats. Based on these results, the authors propose the observed abnormality in *θ*-to-*γ* phase-amplitude coupling as a putative biomarker of A*β*-induced synaptic dysfunction in the early stages of AD development.

L. Jorge et al. correlated A*β* pathology and structural changes between the retinal and primary visual cortex thicknesses in a population of AD patients, by means of integrated imaging techniques. Combining optical coherence tomography, magnetic resonance, and positron emission tomography imaging, the authors measured retinal and primary visual cortex thicknesses, A*β* retention, and neuroinflammation. They report that the primary visual cortex showed increased amyloid-binding potential, in the absence of neuroinflammation, with a positive association between the synapse-rich inner plexiform layers of the retina and the primary visual cortex. This retino-cortical interplay might thus reflect changes in synaptic function resulting from A*β* deposition in AD, even when neuronal loss is still not evident.

Interestingly, to therapeutic prospects based on counteracting mechanisms of A*β*-related synaptic damage, C. Sheng et al. present a study aimed at clarifying the molecular basis of the effects of an empirically used traditional Chinese medicine compound. They characterized the composition of Bushen-Tiansui Formula (BTF), generated a chromatographic fingerprint profile, and evaluated the effects of BTF on cognition and memory functions in a rat model of AD. The oral administration of BTF reversed the cognitive defects by reversing the A*β*_1–42_ fibril-induced reduction of synaptic marker expression and by promoting the expression of Brain-Derived Neurotrophic Factors and the activation of the TrkB/Akt/CREB signaling pathway. This approach points to the importance of accurately defining the potential biological targets of any newly proposed treatment strategy.

Relevant to the early diagnosis of AD, H. Liu et al. explore the possibility of improving the sensitivity of fMRI measures of cortical connectivity that can be used to predict progression from Mild Cognitive Impairment (MCI) to AD. They used the L1-norm linear regression model to test the small-world attributes of the brain networks of three groups of subjects composed, respectively, by 33 MCI, 24 AD, and 27 healthy controls. They found that the L1-norm was more sensitive to detect slight small-world network changes in the early stages of AD with respect to the traditional Pearson correlation analysis.

Y. Li et al. investigated the relationship between Urinary Alzheimer-Associated Neuronal Thread Protein (AD7c-NTP), a marker of neuronal degeneration, and Apolipoprotein Epsilon (ApoE) 4 allele in the cognitively normal population. AD7c-NTP has been shown to be increased in patients with MCI and AD, colocalizing with neurofibrillary tangles and dystrophic neurites. In this study, while no significant differences in urinary AD7c-NTP levels were observed between patients with subjective cognitive decline and healthy controls, AD7c-NTP levels were significantly higher in subjects with ApoE3/4 and 4/4, indicating that also in subjective cognitive decline ApoE4 may modify AD7c-NTP levels, which are related to Tau and A*β* deposition. These findings are extremely relevant for the study of subjective cognitive decline, a stage in which patients report self-experienced persistent decline in cognitive capacity in comparison with a previously normal status, potentially concealing a prodromal state of AD, and that will probably become a target of disease-modifying treatments.

Finally, the advancements in next-generation sequencing (NGS) techniques have allowed for rapid, efficient, and cost-time-effective genetic variant detection. In their article, G. Lanza et al. performed a neurogenetic study through an ad hoc NGS-based custom sequencing gene panel to screen 16 genes in different types of degenerative cognitive disorders. Notwithstanding the preliminary value of the study, some rare genetic variants with a probable disease association were detected, thus suggesting translational perspectives in the diagnosis and management of a wide range of neurodegenerative disorders.

Taken together, findings of the studies in this collection point to the importance of shedding light on the molecular bases and the pathophysiological mechanisms leading to dementia, as this might improve the early diagnostic and prognostic accuracy and therapeutic options.

*Federico Ranieri* *Alberto Benussi* *Mariagiovanna Cantone* *Florinda Ferreri* *Javier Márquez-Ruiz*
